# Epstein–Barr virus-associated lymphomas

**DOI:** 10.1098/rstb.2016.0271

**Published:** 2017-09-11

**Authors:** Claire Shannon-Lowe, Alan B. Rickinson, Andrew I. Bell

**Affiliations:** 1 Institute of Immunology and Immunotherapy, The Medical School, College of Medical and Dental Sciences, University of Birmingham, Edgbaston, Birmingham B15 2TT, UK; 2 Institute for Cancer and Genomic Sciences, The Medical School, College of Medical and Dental Sciences, University of Birmingham, Edgbaston, Birmingham B15 2TT, UK

**Keywords:** Epstein–Barr virus, Burkitt lymphoma, Hodgkin lymphoma, post-transplant lymphoproliferative disease, diffuse large B cell lymphoma, T/NK lymphoma

## Abstract

Epstein–Barr virus (EBV), originally discovered through its association with Burkitt lymphoma, is now aetiologically linked to a remarkably wide range of lymphoproliferative lesions and malignant lymphomas of B-, T- and NK-cell origin. Some occur as rare accidents of virus persistence in the B lymphoid system, while others arise as a result of viral entry into unnatural target cells. The early finding that EBV is a potent B-cell growth transforming agent hinted at a simple oncogenic mechanism by which this virus could promote lymphomagenesis. In reality, the pathogenesis of EBV-associated lymphomas involves a complex interplay between different patterns of viral gene expression and cellular genetic changes. Here we review recent developments in our understanding of EBV-associated lymphomagenesis in both the immunocompetent and immunocompromised host.

This article is part of the themed issue ‘Human oncogenic viruses’.

## 1. Introduction: Epstein–Barr virus infection, virus-induced lymphoproliferative diseases and the virus-associated lymphomas

Epstein–Barr virus (EBV), a human gamma-1 herpesvirus, is widespread in all populations and is carried as a latent asymptomatic infection in the vast majority of individuals. Yet this same agent has powerful lymphocyte growth-transforming ability and is aetiologically linked to a range of lymphoproliferative lesions and malignant lymphomas [1]. Reconciling these two different aspects of its behaviour is still a major challenge and requires a deeper insight into the virus–host interaction.

To summarize current understanding of that interaction, primary infection occurs by the oral route and leads (i) to local virus replication in the oropharynx, involving lytic infection of mucosal epithelium and possibly also local B cells, and (ii) to a virus-driven growth transformation of B cells in pharyngeal lymphoid tissues, followed by a switch to a truly latent (antigen-negative) infection of the generalized memory B cell pool [2]. While these events are most clearly seen during primary infection, they are also on-going during the subsequent carrier state; thus, virus reactivating from the B cell reservoir is thought to seed both new foci of replication in the oropharynx, leading to recurrent low level shedding of infectious virus, and new growth-transforming B cell infections. A large body of evidence suggests that these lytic and growth-transforming latent infections are subject to T cell-mediated immune control both during primary infection and throughout life [3]. Thus, some primary EBV infections are clinically manifest as infectious mononucleosis (IM), a febrile illness characterized by hyper-expansion of both lytic and latent antigen-specific T cell responses, while lower levels of these same responses persist as memory T cells in the blood of all virus carriers and indeed are enriched as tissue-resident populations in oropharyngeal lymphoid tissues where EBV reactivations are thought to initiate [4,5].

Emphasizing the importance of such immune surveillance, individuals with profound T cell impairment are at high risk of developing an acute EBV-positive B-lymphoproliferative disease (B-LPD). The condition is classically seen in two immunocompromised settings: (i) stem cell and solid organ transplant recipients within the first year post-transplant when T cell function is most suppressed (i.e. ‘early onset’ post-transplant LPD), and (ii) HIV-positive patients who, in the era before highly active anti-retroviral therapy (HAART), lost almost all T cell function as they progressed to late stage AIDS. These B-LPD lesions are frequently oligoclonal in origin and can present at multiple sites, often developing within the central nervous system in AIDS patients, leading to their designation as ‘CNS lymphomas’. Cell growth appears to be directly EBV-driven, making B-LPD an *in vivo* counterpart of the B lymphoblastoid cell lines (LCLs) that arise when EBV transforms B cells into permanent growth *in vitro*. Thus the cells have a lymphoblastoid phenotype and express all 8 EBV latent proteins (the nuclear antigens EBNAs 1, 2, 3A, 3B, 3C and –LP and the latent membrane proteins LMPs 1 and 2), a form of infection referred to as Latency III [6]. While B-LPD's frequent oligoclonality and polymorphic appearance has deterred it being designated a true lymphoma, these lymphoproliferative lesions are resistant to conventional cytotoxic drugs and, if untreated, grow to kill the host. Fortunately, however, B-LPD remains highly antigenic and, in a post-transplant setting, will regress if immunosuppression is reduced to allow a recovery of T cell competence or if the disease is specifically targeted by adoptive transfer of EBV latent antigen-specific T cells.

Despite its potent antigenicity, EBV does successfully infect and establish persistence in the naive host. This very likely reflects the ability of some growth-transformed B cells *in vivo* to downregulate latent antigen expression and switch to a truly latent resting state, thereby escaping immune detection. How this occurs is still poorly understood, yet is relevant to the broader question of EBV lymphomagenesis. Thus the fact that all B cell subsets are susceptible to virus infection yet long-term virus carriage is restricted to memory B cells suggests that, initially, virus-transformed cells either pass through a germinal centre (GC) reaction (i.e. exploit the physiologic route whereby antigen-activated B cells somatically mutate their immunoglobulin (Ig) variable gene sequences and progeny with improved antigen avidity are positively selected into B cell memory) or actively generate a GC-like environment and use individual latent cycle proteins at particular phases to mimic the selection process [2]. Whatever the precise details, it seems likely that EBV-infected B cells will enter/re-enter GC reactions either during virus colonization of the B cell system or during their subsequent persistence in the memory pool, and that genetic accidents arising from this normal process will contribute to the pathogenesis of the various EBV-positive B lymphomas [7].

**Figure 1.**
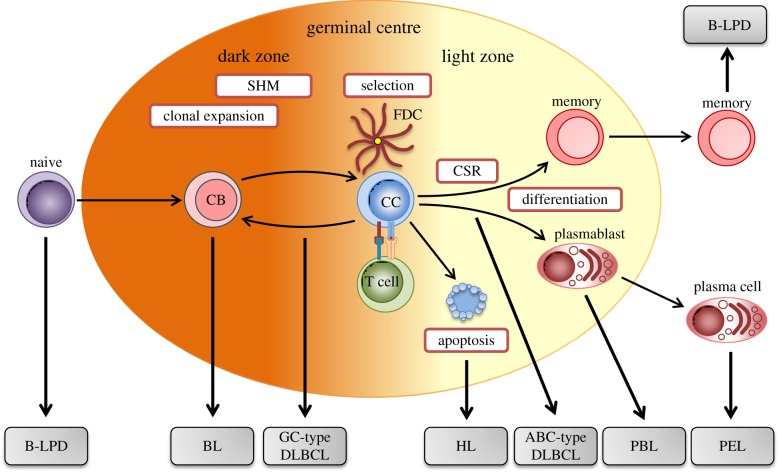
Germinal centre origin of different B cell lymphomas. Circulating naive B cells migrate to the secondary lymphoid organs where, upon encountering antigen, differentiate into centroblasts (CB) that undergo clonal expansion within the dark zone of the germinal centre. During proliferation, the process of somatic hypermutation (SHM) introduces point mutations into the variable region of the Ig heavy and light chain sequences, thereby generating B cells with variant B cell receptors (BCRs). Centroblasts subsequently differentiate into resting centrocytes (CC) and migrate to the light zone, where they are selected on the basis of antigen affinity. Only B cells with advantageous BCR mutations that improve antigen affinity will interact with follicular dendritic cells (FDCs) and receive the appropriate T cell survival signals necessary to evade apoptosis. Antigen-selected B cells can undergo further rounds of proliferation, mutation and selection by recycling to the dark zone. B cells within the light zone can undergo immunoglobulin class switch recombination (CSR), before exiting the germinal centre, either as a memory B cells or plasma cells. Due to the processes of SHM and CSR mediated by activation-induced cytidine deaminase (AID), germinal centre B cells are particularly susceptible to genetic damage. Thus, aberrant AID activity contributes to the chromosomal translocations and mutations that give rise to the different B lymphomas derived from B cells blocked at distinct stages of differentiation. PBL, plasmablastic lymphoma; PEL, primary effusion lymphoma.

The three major types of B cell malignancy linked to EBV are the Burkitt, Hodgkin and diffuse large B cell lymphomas (BL, HL and DLBCL). As illustrated in figure 1, these tumours are thought to emanate from progenitor cells arrested at distinct stages of GC transit or post-GC development. Thus the Burkitt tumour and one subset of diffuse large B cell tumours appear to be derived from germinal centroblasts, whereas the other diffuse large subset and the Hodgkin tumour have hallmarks of post-centroblast cells that have been aberrantly selected later during GC transit. These tumours' relationships to the GC, inferred from tumour cell phenotype and the presence of somatically mutated Ig variable genes, emphasize the likely contribution that genetic aberrations occurring within the GC have made to tumour development. By contrast, the classical EBV-driven B-LPD lesions seen early post-transplant are not GC-derived but arise from virus-induced growth transformation of either naive or mature memory B cells [8]. Recent work suggests that naive B cell-derived lesions are more commonly seen following stem cell transplant [9]. This may reflect the fact that stem cell recipients often acquire or reacquire EBV in the peri-transplant period when the repopulating B cell pool is dominated by naive cells, whereas solid organ (mainly kidney) graft recipients are typically already long-term EBV carriers pre-transplant and disease may arise from reactivation of existing memory cell infection. While the early onset post-transplant B-LPDs are always EBV-positive, the three major EBV-associated lymphomas, and most of their subtypes, can occur in EBV-positive or negative form. This is particularly important because it suggests that, for each tumour, there are at least two routes to a common end, only one of which involves EBV infection. Indeed, comparisons between EBV-positive and -negative tumours of the same subtype, especially with respect to the landscape of cellular genetic change, has great potential to identify those genomic changes that EBV infection renders redundant.

Table 1 summarizes the essential characteristics of the EBV-associated B lymphomas and their subtypes, including their degrees of EBV association, and their distinct patterns of EBV latent antigen expression. For ease of presentation these patterns are designated Latencies I (EBNA1 only), II (EBNA1, LMP1 and LMP2) or III (full latent gene expression), though it should be remembered that this classification is not always hard and fast; in particular, tumours expressing EBNA1 and low levels of LMP2 blur the boundary between Latencies I and II, while different tumour types designated Latency II can differ significantly in LMP1 and/or LMP2 protein levels (see table 1 footnote for further qualification). These different latency programmes have clear implications for the visibility of tumours to the T cell system and bring into question the relationship between lymphoma risk and host immune status. An absolute dependence upon immune impairment is clear in the case of EBV-driven B-LPD lesions, where EBV-transformed cells grow out opportunistically in the absence of T cell control. However, the situation with respect to the three main EBV-associated B cell lymphomas is more complex. On the one hand most of these lymphomas, including those that are EBV-positive, arise in individuals who are apparently immunocompetent, implying the EBV-positive tumour's independence from virus-specific immune surveillance. On the other hand, immune disturbance does affect lymphoma risk; thus, the incidence of all three lymphomas, and the proportion of those tumours that are EBV-positive, is increased in HIV-positive individuals. A similar, but less marked, trend is also seen in long-term transplant recipients. However, in contrast to the acute B-LPDs, the heightened lymphoma risk in these patient groups is not coincident with times of peak T cell impairment and other factors must come into play. Besides these B cell diseases, table 1 also lists the surprising links between EBV, ostensibly a B-lymphotropic virus, and both lymphoproliferative disease and malignant lymphomas of T or NK cell origin. These less well studied diseases bear witness to the rare occasions in which EBV gains fortuitous access to the T- and/or NK-cell lineages, with serious consequences for the host as described in the later sections of this review.

**Table 1.** Features of EBV-associated lymphomas.

disease	cellular origin	% EBV association	EBV latency^a^	latent EBV protein expression^b^
B-lymphoproliferative disease				
post-transplant	naive or memory	>90	III	EBNAs 1, -2, -3A, -3B, -3C, -LP, LMPs 1, 2
HIV-related	B cell	>90		
Burkitt lymphoma				
endemic		100		
sporadic	GC centroblast	10–80	I	EBNA1
HIV-related		30–40		
classical Hodgkin lymphoma				
nodular sclerosis		10–40		
mixed cellularity		70–80		
lymphocyte depleted	post-GC centroblast	10–50	II	EBNA1, LMPs 1, 2
lymphocyte rich		30–60		
HIV-related		>90		
diffuse large B cell lymphoma				
NOS		10	II/III	EBNA1, LMPs 1, 2/all EBNAs, LMPs 1, 2
PAL	post-GC centroblast	100	III	EBNA1, -2, -3A, -3B, -3C, -LP, LMPs 1, 2
HIV-related		20–60	I/II/III	EBNA1/EBNA1, LMPs 1, 2/all EBNAs & LMPs
rare immunocompromised B lymphomas				
plasmablastic lymphoma	plasmablast	75–90	I	EBNA1
primary effusion lymphoma	plasma cell	75–90	I	EBNA1
T/NK lymphoproliferations				
CAEBV	T/NK/B	100		
extra-nodal T/NK lymphoma	T/NK	100	II	EBNA1, LMPs 1, 2
aggressive NK lymphoma	T/NK	100		

^a^For convenience, the patterns of EBV latent gene expression are frequently designated Latency I (EBNA1 only), Latency II (EBNA1, LMP1 and LMP2) or Latency III (EBNAs1, -2, 3A, -3B, -3C, -LP, LMP1 and LMP2). However, it is now clear that disease-associated viral signatures may be more complex than originally described. For example, Latency I and Latency II are now considered to represent opposite ends of a spectrum with variable degrees of expression of both LMP1 and LMP2. In addition, transcriptional studies have recently identified broader patterns of viral gene expression in which latently infected cells also express varying levels of multiple lytic cycle transcripts. While these findings imply that a restricted set of viral lytic genes may be transcribed during an otherwise latent infection, it remains unclear whether these additional lytic viral transcripts are translated or if they are expressed in every tumour cell.

^b^In addition to latent protein expression, EBV-infected cells also express a series of non-coding viral RNAs. The EBER1/EBER2 transcripts and 44 mature miRNAs derived from the BamHI A rightward transcripts (miR-BARTs) are present in all forms of latency, while 4 further BHRF1-derived miRNAs are expressed in Latency III.

## 2. Burkitt lymphoma

Burkitt lymphoma (BL) is a highly aggressive non-Hodgkin B cell tumour that can be classified into three variants based on clinical features and disease epidemiology [10]. Endemic BL (eBL), described by pioneering studies of Dennis Burkitt, affects young children in regions of equatorial Africa and Papua New Guinea, where it accounts for around 50% of all paediatric cancers. This high incidence form of the disease classically presents as a jaw tumour and is EBV-positive in almost every case [11]. From epidemiological studies, it is clear that eBL is restricted to geographical regions where *Plasmodium falciparum* malaria is holoendemic and, coincidentally, where primary EBV infection occurs at a young age [12]. By contrast, sporadic BL (sBL) occurs worldwide, has a much lower incidence and affects children across a slightly older age range. In the United States and Europe, sBL is rarely associated with EBV but in other areas, notably NE Brazil where BL incidence appears to be higher, EBV rates can exceed 80% [13]. A third variant (more appropriately termed ‘HIV-related BL’ than its usual description as ‘AIDS-related’) affects HIV-infected individuals. From studies of the AIDS epidemic in the developed West, HIV infection increases BL incidence by more than 100-fold above that of the sporadic disease, and some 30–40% of these tumours are EBV-positive. This led to BL being designated an AIDS-defining illness; importantly, however, the tumour typically develops early in the course of HIV infection, coincident with symptoms of persistent generalized lymphadenopathy and before circulating CD4^+^ T cell numbers begin to fall [14]. The contrast between this tumour and the EBV-positive B-LPD lesions seen in late stage AIDS is further emphasized by looking at incidence rates before and after the introduction of highly active anti-retroviral drug therapy in the late 1990s. Thus, while the numbers of B-LPD lesions (including those recorded as primary CNS lymphomas) have fallen dramatically in the post-HAART era, the incidence of HIV-related BL has not changed [15–17].

Regardless of subtype and EBV status, all BL tumours are morphologically and immunophenotypically similar, and share a common gene expression signature resembling that of centroblasts [18,19]. The tumours are composed of a monomorphic population of rapidly proliferating medium-sized B cells characterized by the expression of IgM, CD10 and BCL6, and bearing functionally rearranged and somatically mutated Ig sequences indicative of a germinal centroblast origin. Histologically, they are largely devoid of normal infiltrating lymphocytes with the exception of scattered phagocytic macrophages, which give rise to the characteristic starry sky appearance. Another key defining feature of all three BL variants is a chromosomal translocation which juxtaposes the c-MYC oncogene on chromosome 8 and one of the three Ig loci, most frequently the Ig heavy chain locus on chromosome 14 [20]. As a consequence of bringing the c-MYC gene under the influence of the powerful Ig enhancer, BL cells constitutively express high levels of MYC protein leading to uncontrolled cell growth [21]. MYC overexpression also contributes to maintaining the centroblast phenotype of BL tumour cells by promoting a GC-like gene expression programme [22]. Importantly, however, studies from Eµ-MYC mouse models suggest that MYC overexpression is not sufficient to drive lymphomagenesis, in part, because high levels of MYC induce a powerful p53-mediated apoptotic stress response which ultimately must be overcome during tumourigenesis. To counteract this apoptosis, the majority of BL cell lines and around 30% of BL tumours carry TP53 mutations, while BL tumours that retain wild-type p53 carry mutations or epigenetic changes that lead to aberrant expression of the p53 regulator proteins, p19/ARF and MDM2 [23]. In addition, the translocated c-MYC allele is itself frequently mutated, thereby subtly altering its transactivation capacity and reducing its ability to promote apoptosis.

As a result of several groundbreaking genetic studies, we are now beginning to understand the additional oncogenic events that cooperate with c-MYC deregulation during BL pathogenesis [24–28]. To date, recurrent mutations have been identified affecting genes related to cell cycle (e.g. CCND3, CCNF, CDKN2A), apoptosis (e.g. MCL1) and chromatin remodelling (e.g. ARID1A, SMARCA4), as well as genes implicated in other lymphomas (e.g. DDX3X). Although the functional relevance of many of these changes remains to be determined, studies have revealed that oncogenic CCND3 mutations prevent the normal physiologic regulation of cyclin D3 protein levels, leading to an accumulation of cyclin D3 protein [24]. Notably the incidence of such CCND3 mutations is reportedly much higher among sBL than eBL tumours, hinting at different pathogenetic mechanisms in these two subtypes [29]. Furthermore, up to 70% of BL cases carry genetic lesions that stimulate the activity of TCF3 (E2A), a transcription factor that functions as master regulator of gene expression in GC centroblasts. One important consequence of increased TCF3 activity is amplified ‘tonic’ (antigen-independent) B cell receptor (BCR) signalling and activation of the pro-survival PI3K/AKT pathway, which may be reinforced by additional mutations or the expression of cellular miRNAs that abrogate PTEN expression [29]. The critical role of PI3K signalling in BL pathogenesis is further emphasized by the finding that activated PI3K cooperates with MYC during lymphomagenesis [30]. Interestingly, this dependency of BL tumours on tonic BCR signalling may explain why, even though one Ig allele is involved in the c-MYC translocation, all BLs retain surface Ig expression from a second functionally rearranged allele.

While EBV is clearly not essential for the development of BL, an aetiological role for virus infection is supported by the finding that in EBV-positive cases every tumour cell harbours monoclonal EBV genomes. However, there is still much debate regarding the precise details of BL pathogenesis in terms of the nature of the B cell initially infected with EBV, whether EBV infection precedes or follows the c-MYC translocation, and which viral genes are involved at different stages of the malignant transformation process [21,31,32]. In this regard, viral gene expression in most EBV-positive BL tumours is restricted to EBNA1, the non-coding EBER RNAs and a cluster of 45 BART-miRNAs (table 1), with LMP2 expression recently detected in a small subset of tumours [17,33]. Since these viral gene products *per se* are insufficient to induce B cell proliferation, it is proposed that EBV has other accessory roles during BL pathogenesis. One view is that EBV increases the survival of BL tumour cells following the c-MYC translocation event, as evidenced by the original finding that EBV-positive BL cell lines are more resistant to apoptosis than isogenic EBV-negative counterparts [34]. However, there is still no clear consensus on the mechanism underpinning this observation, with some reports attributing these effects to the oncogenic EBER RNAs [35] while others suggest roles for EBNA1 and the BART-miRNAs. In this regard, EBNA1 has recently been shown to deregulate multiple cellular genes implicated in B cell survival [36,37] and may prevent cell death through interactions with host proteins such as the anti-apoptotic protein survivin [38] and the p53-regulator USP7 [39]. In recent years, attention has switched to identifying potential functions for the multiple BART-miRNAs. There is now strong evidence that these viral miRNAs are not only critical for optimal EBV-induced B cell growth transformation [40], but also play a key role in maintaining the BL phenotype [41–43]. However, with the notable exception of caspase-3 [41,44], few direct targets of EBV BART-miRNAs relevant to BL survival have been identified to date.

An alternative (but not mutually exclusive) scenario is that EBV promotes the likelihood of a c-MYC translocation event within a GC B cell as a result of aberrations during somatic Ig gene mutation and subsequent Ig class switch recombination (see figure 1 legend). This is supported by the finding that activation-induced cytidine deaminase (AID), the enzyme that mediates both of these processes, also promotes the generation of Ig/c-MYC translocations [45]. Notably EBV infection induces AID activity [46], although a recent study concluded that this effect is mediated by EBNA3C [47], a viral gene product not usually expressed in BL tumours. One possible explanation to this paradox is that early BL progenitors resemble EBV-transformed B lymphoblastoid cells (i.e. Latency III infection), and that the subsequent transition to a more restricted Latency I pattern seen in the final tumour occurs after the c-MYC translocation and acquisition of additional compensatory genetic changes [21,31]. Consistent with this idea are recent data suggesting that activation of c-MYC transcription by the viral transactivator EBNA2 predisposes the upstream c-MYC enhancer region to AID-induced double-strand DNA breaks [48]. Holoendemic malaria, which is tightly associated with the African endemic form of the disease, has also been shown to induce AID activity while simultaneously increasing the number of EBV-infected cells within the GC [49,50]; these observations thus provide the first mechanistic link to explain how malaria co-infection predisposes EBV-positive B cells to acquire a c-MYC translocation. Likewise, as discussed elsewhere [51], the early stages of HIV infection are associated with persistent generalized lymphadenopathy, reflecting a huge expansion of GC activity within lymphoid tissues, and a shift towards a higher EBV load in the B cell system. Respectively, these two factors likely underpin the marked increase in BL incidence seen in HIV-positive cohorts in Western countries and (compared to the low EBV-association rates shown by sBL in these areas) the much higher proportion of these HIV-related tumours that are EBV-positive.

Despite these recent advances, our understanding of BL pathogenesis remains incomplete and, in particular, it is unclear whether BL variants may arise by different pathogenetic routes. In this regard, eBL and sBL differ in the detailed anatomy of Ig/c-MYC breakpoints [52], in the frequency and pattern of Ig mutations [53] and in their gene expression profiles [54], implying that EBV-positive and negative forms of the disease may originate from GC B cells at subtly distinct stages of maturation. There are also differences in the mutational landscape between eBL and sBL cases [29], and, as recently reported, between BL tumours carrying different EBV strains [55] suggesting that EBV compensates for the lack of certain cellular mutations. However, further studies will be required to determine the functional significance of the above differences.

## 3. Hodgkin lymphoma

On the basis of distinct morphological and clinical features, Hodgkin lymphoma (HL) can be divided into classical HL (cHL), which accounts for the majority of cases, and nodular lymphocyte predominant HL (NLPHL) [10]. Since NLPHL is rarely EBV-positive, here we will focus on cHL where the link with EBV is well established. As shown in table 1, cHL can be further subdivided into four histological subtypes: nodular sclerosis, mixed cellularity, lymphocyte depleted and lymphocyte rich [56], of which the first two subtypes account for approximately 90% of all cHL cases. A common feature of all four subtypes is that the neoplastic Hodgkin and Reed-Sternberg (HRS) cells account for only 1–2% of the tumour mass [57]. This paucity of HRS cells has hindered both the clarification of their cellular origin and the identification of genetic lesions that contribute to HL pathogenesis. The remaining cells comprise a non-neoplastic cell infiltrate composed of T cells, B cells, macrophages, eosinophils and fibroblasts. As reviewed elsewhere, crosstalk between this tumour microenvironment and the malignant HRS cells contributes to the growth, survival and immune escape of the tumour [58]. The proportion of cHL cases associated with EBV varies dramatically with age, gender, ethnicity, country of residence and histological subtype [59]. EBV association rates in developed countries range between 30% and 50%, but much higher rates have been reported in developing countries. EBV-positive cases are more prevalent in children under 10 years and adults over 80 years, and are usually associated with the less common mixed cellularity and lymphocyte-depleted subtypes. Given the association between cHL and infectious mononucleosis, cHL arising in children and young adults may be a rare consequence of primary EBV infection, while the peak in older adults may be attributed at least in part to senescence of EBV immunity and an increasing EBV load.

Particularly interesting in this regard are the data from HIV-infected cohorts. Even though HL is not an AIDS-defining illness, the incidence of cHL is 10-fold higher than in the general population, and essentially all of that increase involves EBV-positive tumours of mixed cellularity or lymphocyte-depleted subtypes [60]. Just as with BL, the development of HIV-related HL is not dependent on profound T cell impairment; indeed cHL incidence among HIV carriers has slightly increased after the introduction of HAART [16]. Interestingly these tumours tend to arise somewhat later than HIV-related BL, in patients with relatively modest reductions in CD4^+^ T cell counts. One possibility is that partial CD4 suppression is sufficient to elevate EBV loads in the B cell system while retaining the cytokine-mediated T cell support through which CD4^+^ T cells may contribute to cHL pathogenesis.

The presence of clonally rearranged and somatically mutated Ig sequences in microdissected tumour cells reveals that HRS cells are derived from GC or post-GC B cells [61]. However, HL is unique among B cell lymphomas in that neoplastic HRS cells have largely lost their B cell identity during the process of malignant transformation. This phenomenon is partly due to the repression of key B-cell-specific transcription factors, such as TCF3 and EBF1, leading to a global downregulation of B cell lineage gene expression [62,63]. Recent data suggest that re-expression of TCF3 induces apoptosis and cell-cycle arrest in cHL cell lines, indicating that TCF3 is an important tumour suppressor gene in the context of cHL [64]. This notion is supported by the results of a recent genome-wide association study which identified a TCF3 polymorphism linked to an increased risk of HL [65]. Another hallmark of HRS cells is the loss of a functional BCR as a result of epigenetic silencing of the Ig promoter, repression of B cell transcription factors and loss of downstream components of the BCR signalling pathway [57,66]. In addition, around 25% of HRS cells carry destructive Ig mutations that render the BCR non-functional [67]. While B cells lacking a functional BCR arise through the normal physiologic process of SHM, they would usually be rapidly eliminated by apoptosis. Thus, HRS cells are likely derived from ‘crippled’ pre-apoptotic GC B cells that have been rescued by additional transforming events (figure 1)

A key pathogenic feature of HRS cells is the aberrant activation of numerous cell signalling pathways and transcription factors that support the growth and survival of the tumour. Extensive studies of cHL cell lines and primary biopsies have identified two critical cell signalling pathways, NF-κB and JAK/STAT, which are frequently deregulated in HRS cells. Constitutive NF-κB signalling results from the expression of multiple tumour necrosis factor (TNF) receptors, such as CD30, CD40 and RANK, which are activated by the cellular infiltrate surrounding the HRS cells [58]. NF-κB activity is further enhanced by a variety of genetic lesions in HRS cells, including amplification of the c-REL subunit, loss of inhibitory IκB subunits and inactivation of TNFAIP [68]. JAK/STAT activation can be triggered by autocrine and paracrine growth factors, including IL-7, IL9 and IL-13 [58] and these signals can be intensified by mutations in the JAK/STAT pathway [69]. However, our broader understanding of the transforming events that contribute to HL pathogenesis remains limited, due in part to the rarity of HRS cells within biopsy material, although modern sequencing technologies combined with improving methods of tumour cell selection are now beginning to yield new insights [70,71]. One recent exome analysis using flow-sorted HRS cells identified 99 recurrently mutated genes; while some of these genes had been described in earlier targeted sequencing studies, others identified novel genes not previously linked to lymphoid malignancies. Notably, truncating mutations of beta-2-microglobulin (B2M) were strongly associated with the nodular sclerosis subtype and younger age at diagnosis [70]; however it remains controversial whether disruption of B2M/MHC class I expression leads to a better clinical outcome [70,72].

Although it has long been recognized that a proportion of cHL tumours harbour monoclonal EBV genomes, we still do not fully understand how the virus contributes to disease pathogenesis. In this regard, EBV-infected HRS cells express three viral proteins, EBNA1, LMP1 and LMP2, together with the non-coding EBERs and BART miRNAs, the classical Latency II pattern (table 1). Of these, the uniquely high levels of LMP1 seen in EBV-positive HRS cells seem likely to be essential in the pathogenesis of HL, since LMP1 activates the NF-κB, JAK/STAT and PI3K cell signalling pathways, and induces transcriptional changes in GC B cells that are characteristic of HRS cells [73]. Another potential role for LMP1 may involve suppression of the virus lytic cycle that might otherwise lead to cell death. In *in vitro* models, the switch from latency to virus replication in EBV-infected B cells can be triggered by plasma cell differentiation or activation of BCR signalling. Recent data suggest LMP1 may inhibit the former by disrupting the BLIMP1 transcription programme necessary for terminal B cell differentiation [74], while the latter may be blocked by the loss of a functional BCR. By contrast, the contribution of LMP2 to HL is less well understood. LMP2 signalling can provide a survival signal through engaging the PI3K pathway and recapitulate some of the gene expression changes seen in EBV-positive HRS cells [75]. These changes are mediated by the N-terminal ITAM motif of LMP2 which resembles the signalling domain of the BCR, yet many of the downstream signalling components necessary for both BCR and LMP2 signalling are absent in HRS cells. Thus, one possibility is that LMP2 activity is only required in early HRS progenitor cells which retain the downstream BCR signalling molecules, but that LMP2 function is subsequently replaced by additional genetic changes in the malignant HRS cells. However, given that EBV-positive cases of HL are consistently LMP2-positive, it is likely that LMP2 also has BCR-independent functions that are important for maintenance of the HRS phenotype.

Although EBV-positive and EBV-negative cases of cHL appear morphologically and phenotypically similar, there is increasing evidence that these two entities arise through different pathogenetic routes. First, nearly all cases of cHL carrying crippled BCRs are EBV-positive suggesting that HRS precursors with such mutations can only survive and undergo malignant transformation if infected with EBV [76]. In this regard, EBV can rescue BCR-negative GC B cells from apoptosis [77]. Second, TNFAIP3 mutations and aberrant expression of multiple receptor tyrosine kinases (RTKs) appear to be more common in EBV-negative cases [78,79], indicating that EBV functionally substitutes for certain deregulated cell signalling pathways in HRS cells. Finally, EBV infection has been shown to induce epigenetic and transcriptional changes in primary GC B cells relevant to HL [80]. Therefore, it appears that the aetiological role for EBV is to provide the necessary signals required for the growth and survival of the tumour by compensating for the lack of critical cellular mutations, in particular the absence of a functional BCR, in the HRS progenitor cells.

## 4. Diffuse large B cell lymphoma

Diffuse large B cell lymphoma (DLBCL) is the most common type of high-grade non-Hodgkin lymphoma in adults, accounting for up to 40% of cases globally. DLBCL is a heterogeneous disease, originally classified by the WHO in 2008 as two molecular subtypes, based upon microarray-based gene expression profiling [81]; germinal centre B-cell-like (GCB) DLBCL and activated B-cell-like (ABC) DLBCL. Subsequent analysis by next generation sequencing (NGS) has confirmed that GCB and ABC DLBCLs have unique gene expression signatures as well as different patterns of genetic mutation. This has led to the further classification of the two major subtypes of DLBCL into more clearly delineated diseases [10].

The 10% of DLBCLs that are EBV-positive (i.e. carrying the virus genome in every malignant cell) constitute one particular group. Such tumours, typically lying within the spectrum of ABC DLBCLs, were originally identified in older patients and provisionally termed EBV-positive DLBCL of the elderly [82,83], being defined as any EBV-positive monoclonal large B cell lymphoproliferation in an immunocompetent patient greater than 50 years old. However, the disease has subsequently been re-classified as DLBCL-NOS (‘not otherwise specified’) to take into account the increasingly documented occurrence of EBV+ DLBCL in younger immunocompetent patients [10,84,85]. The classical EBV+ DLBCL observed in the elderly is thought to arise as a consequence of an age-related senescence of EBV-specific T cell surveillance. The precise nature of the senescence-associated defects in T cell immunity have not yet been well defined, although a narrowing of the EBV-specific TCR-Vβ repertoire with reduced EBV-specific effector memory CD4^+^ and CD8^+^ T cell numbers has been described [86]. Consistent with reduced T cell immunity, these tumours usually display a Latency III viral gene expression profile [87], similar to that seen in the B-LPD lesions arising in immunosuppressed transplant recipients or in late-stage AIDS. In this regard, a recent study has indicated that the EBV-encoded nuclear antigen EBNA3B, which is dispensable for virus-driven B cell transformation, may actually attenuate the oncogenic potential of EBV. Thus, inactivation of EBNA3B promoted more aggressive DLBCL-like monomorphic tumours in mice reconstituted with a human immune system. B cells infected with EBNA3B-deleted virus proliferated more rapidly and secreted less T cell chemoattractant CXCL10, thereby reducing T cell recruitment at the tumour site [88]. Notably, the same study also identified a number of tumour-associated EBNA3B mutations although the functional effects of these changes were not investigated. In contrast, EBV+ DLBCL arising in younger patients usually displays a Latency II gene expression profile, implying a more complex multi-step pathogenesis involving some form of immune evasion. Note that tumours of the ABC DLBCL subtype generally have a worse outcome than the GCB subtype, and this is further exacerbated by EBV infection, with EBV+ DLBCL-NOS cases showing a higher rate of extranodal involvement, a more aggressive clinical course and frequent refractory disease.

In molecular terms, EBV+ ABC DLBCL cells frequently exhibit monoclonality by Ig gene rearrangements and by analysis of EBV terminal repeat copy number. Just as in EBV-positive BL and HL therefore, this strongly supports involvement of EBV in the process of lymphomagenesis, complementing the effects of cellular genetic changes. As to the nature of those cellular changes, cytogenetic analysis of EBV+ DLBCL-NOS has not identified characteristic abnormalities, although copy number gains have been reported of the c-MYC, BCL2 and BCL6 loci [89]. However, fewer chromosomal alterations have been demonstrated for the EBV+ ABC DLBCL than the EBV-negative counterparts, again supporting a significant role for EBV in driving pathogenesis. Exactly what that role might be remains to be determined but there are important hints coming from the wider genetic profiling of ABC DLBCLs. Biochemical studies have now demonstrated the reliance of ABC DLBCL on genetic lesions leading to constitutive NF-κB activation and chronic active BCR signalling [90]. Constitutive NF-κB activation helps to explain two key characteristics of these tumours, their plasmablastic phenotype and frequent resistance to chemotherapy. Firstly, in the normal B cell differentiation pathway, NF-κB stimulates its target gene IRF4, which in turn transactivates PRDM1/Blimp-1 to induce full plasma cell differentiation. While NF-κB signalling is constitutively active, most ABC DLBCLs carry either inactivating point mutations, deletions or epigenetic silencing of the PRDM1 gene, and consequently tumour cells are driven to accumulate at the plasmablast stage [91–93]. Secondly, NF-κB dysregulates several pro-survival proteins, including BCL-XL, cIAP1, cIAP2 and c-FLIP, potentially resulting in chemotherapy-refractory disease [94]. In this context, the EBV+ DLBCLs have an even more pronounced NF-κB activation phenotype compared to the bulk of EBV-negative DLBCLs, with nuclear expression of both the canonical and non-canonical NF-κB pathways. This is thought to reflect activation by LMP1, clearly expressed in most, if not all, EBV+ DLBCLs, implying that the presence of EBV in these tumours contributes to their refractory nature. Only the constitutive NF-κB activation phenotype of this EBV+ DLBCL subset has been examined while the role of chronic BCR signalling is yet to be resolved. To date, only one gene expression profiling study has been performed specifically comparing EBV+ with non-EBV-associated ABC DLBCL [95]. This identified high levels of expression of immune and inflammatory gene pathways in the EBV+ tumours including, in addition to NF-κB, the JAK/STAT, NOD receptor and Toll-like receptor signalling pathways. Importantly, *in vitro* studies of EBV infection of ABC DLBCL cell lines confirmed the enhanced activation of NF-κB and increased phosphorylation of STAT3 was induced by EBV infection [95].

An unusual subset of DLBCLs comprises EBV-positive tumours that occur in the context of long-standing chronic inflammation. These tumours are also ABC DLBCL, derived from late GC or post-GC B cells. These DLBCLs usually present as a tumour mass involving body cavities, and pyothorax-associated lymphoma (PAL) is the prototypic form. PAL is associated with a history of chronic pyothorax or chronic pleuritis due to the initiation of a therapeutic artificial pneumothorax for pleuropulmonary tuberculosis, which was used in the past as surgical treatment for tuberculosis [96]. The interval between the onset of chronic inflammation and malignant lymphoma is usually over 10 years (median 37 years), with a median patient age of 65–70 years. Males are more susceptible to this lymphoma, with a male to female ration of 4 : 1. Other EBV-positive DLBCLs associated with chronic inflammation and resembling PAL arise in the context of metallic implants in bones and joints [97], of chronic osteomyelitis, and of chronic venous ulcers [98,99]. The EBV gene expression profile of PAL is usually Latency III. It has been suggested that the EBV-transformed B cells at the site of chronic inflammation are able to escape host immune surveillance and grow out through several mechanisms, for example involving production of cellular IL-10, an immunosuppressive cytokine [100,101], autocrine growth promotion via the IL-6 and IL-6 receptor pathway [102], downregulation of MHC class I expression [103] and mutation of immunodominant T cell epitopes in EBNA3B [104]. In addition, microarray analysis has identified interferon-inducible 27 (IFI27) as one of the most differentially expressed genes in PAL compared to regular DLBCL [105]. Expression of IFI27 is in keeping with the role of chronic inflammation in this condition since it is known to be induced in B lymphocytes following stimulation with IFNα, although the role of IFI27 in lymphomagenesis has yet to be resolved.

DLBCLs that arise with increased incidence in HIV-positive patients are another very instructive subset. In the early years of the AIDS epidemic, reports of HIV-related DLBCLs were dominated by tumours that were in effect B-LPD lesions of late stage AIDS yet recorded as immunoblastic (i.e. ABC-like) DLBCL; accordingly, they were described as predominantly (90%) EBV-positive with a Latency III infection. Following the advent of HAART, the incidence of such late-stage lesions has dropped to reveal an underlying landscape of both non-GC-like and GC-like centroblastic tumours that can appear at any stage of HIV infection [106]. Current surveys show that, overall, some 30–35% of these DLBCLs are EBV-associated and, among these, there is a greater spread of latency programmes than was previously apparent [60]. Thus a recent study found that 27/48 (56%) HIV-related DLBCLs of non-GC type were EBV-positive with almost equal representation of Latency I, Latency II and Latency III tumours, whereas in the larger group of GC-like DLBCLs, 25/98 (25%) were EBV-positive and there were predominantly of Latency I [17]. The composition of HIV-related DLBCLs has therefore changed post-HAART, but importantly the overall incidence and EBV association rate of these tumours still remains significantly higher than for DLBCL in the general population.

A somewhat similar picture is now being recognized among solid organ transplant recipients who, following an initial period of high dose immunosuppression, are maintained on low level immunosuppressive therapy for many years. While their risk of EBV-positive B-LPD is most acute in the first year and proportional to the intensity of immune suppression, these patients also carry an increased risk of post-transplant lymphoma, particularly DLBCL, throughout life [107,108]. Recent studies of such ‘late onset’ tumours suggest that 30–70% are EBV-positive, with Latencies I, II and III all represented among the EBV-positive subset [9,109–111]. There were also interesting differences between the tumours based on EBV status. Thus the EBV-positive DLBCLs had fewer genetic aberrations than their EBV-negative counterparts, including fewer mutations affecting NF-κB pathway genes, [109,111], had distinct transcription profiles [110,112], and displayed markers of a more immunosuppressive local environment [9]. Based on such findings, it is clear that the pathogenesis of these ‘late onset’ post-transplant tumours is more complex than that of ‘early onset’ B-LPD; however, where the virus is present, it has influenced the genomic evolution of these tumours and continues to influence the DLBCL phenotype.

## 5. Other rare B lymphomas of the immunocompromised state

For completeness, table 1 also includes two additional EBV-associated tumours of B cell origin, plasmablastic lymphoma (PBL) and primary effusion lymphoma (PEL). Both are rare malignancies that were first recognized in late-stage AIDS patients [113,114] but have since been seen occasionally in other heavily immunocompromised settings [60,115]. PBLs, identifiable through a distinct plasmablastic phenotype, typically present in the oral cavity but can appear at other sites; PELs present as lymphomatous effusions where (as in HL) the malignant cells have the Ig genotype of post-GC B cells but have downregulated B cell markers. In both cases, the majority (75–90%) of tumours are EBV-positive with Latency I infection predominant. However, the virus' contribution to the pathogenesis of these tumours is unclear. The question is of particular interest in the case of PELs, tumours which are consistently positive for the Kaposi sarcoma-associated herpesvirus (KSHV) genome and express KSHV latent proteins [116]. Current evidence comparing EBV-positive and negative PELs shows that the virus does subtly influence the PEL cell phenotype [117], and so EBV may accelerate KSHV-mediated lymphomagenesis either by directly impacting on cell growth and apoptosis pathways or, alternatively, by facilitating the maintenance of latent KSHV infection.

## 6. Epstein–Barr virus-associated T/NK cell lymphoproliferative diseases and malignancies

EBV is a B-lymphotropic virus that does not infect the T- and NK-cell lineages as part of its natural life cycle *in vivo*. However, ectopic T and/or NK cell infection does occur on very rare occasions, leading either to EBV-driven lymphoproliferations (EBV+ T/NK LPD), clinically manifest as chronic active EBV infection (CAEBV), or to highly aggressive monoclonal malignancies such as extranodal NK/T cell lymphoma (ENKTL) and aggressive NK leukaemia (ANKL). While these frankly malignant lymphomas may arise de novo, others develop from a scenario of chronic active infection. Indeed, up to 25% of the CAEBV cases will progress to malignancy. Due to the paucity of information on ANKL, here we will discuss the T/NK associated LPDs and malignancies, with particular emphasis on CAEBV and ENKTL.

### (a) Chronic active Epstein–Barr virus infection

CAEBV is usually described as a chronic disease of childhood and young adulthood where patients exhibit persistent IM-like symptoms including fever, hepatosplenomegaly, persistent hepatitis and extensive lymphadenopathy. This is coupled with elevated EBV DNA load in the peripheral blood mononuclear cells, histological evidence of organ infiltration with EBV-infected cells and high concentrations of pro-inflammatory cytokines in the blood. Although CAEBV is usually considered a chronic disease, many patients develop severe, often fatal complications including multi-organ failure, digestive tract ulceration/perforation, and haemophagocytic lymphohistiocytosis (HLH), in addition to the risk of progression to T/NK malignancy.

Worldwide, CAEBV exhibits a strong ethnic and geographical predilection with the highest frequency of cases in East Asia and in native populations of Central/South America and Mexico, themselves of Asian origin. The majority of cases occur in children and young adults of either sex with a mean onset of 11.3 years [118]. The frequency of CAEBV arising from T cell versus NK cell infection is roughly equal, but there are some clinical differences. Thus NK cell CAEBV is usually milder and slowly progressing, though with a surprisingly higher viral load [119], whereas T cell CAEBV is more severe and rapidly progressive with a higher incidence of hepatomegaly and lymphadenopathy [120]. It remains unclear why this should be. Interestingly, the number of cases of CAEBV in adults appears to be increasing, perhaps because of greater awareness among clinicians; adult cases predominantly (87%) arise from T cell infections and are often rapidly progressive.

The first case of CAEBV was described in 1978 yet its pathogenesis is still poorly understood. Monoclonality of the lymphoproliferative lesion by cellular and EBV terminal repeat markers [121,122] strongly suggests its origin from a single EBV-infected cell. However, no consistent mutations or chromosomal aberrations have been identified in studies to date [123–125]. The infected cells exhibit a Latency II viral gene expression profile (table 1), including the newly identified LMP2-TR transcript [126]. Despite this expression of viral antigens*,* the infected cells appear to evade immune surveillance and/or profit from impaired surveillance *in vivo*. Many patients exhibit impaired NK cell and EBV-specific CTL activity [124,127], but it is unclear if this is a cause or consequence of the disease process. A consistent feature of CAEBV is the elevated plasma levels of both pro- and anti-inflammatory cytokines, including interleukin (IL)-1β, interferon (IFN)-γ, IL-10, IL-13, IL-15, TNF-α and transforming growth factor (TGF)-β [120,128]. Interestingly, the cytokine profiles of T cell and NK cell CAEBV are surprisingly similar, with the exception of IL-13 which is significantly higher in NK cell cases. Given IL13's involvement in IgE responses, this could help to explain the high serum IgE levels and hypersensitivity to mosquito bites frequently observed in NK cell CAEBV patients.

Individuals with the symptoms of CAEBV have also been well documented in the US, but they differ from those observed in East Asia in several respects. In many cases the EBV infection is confined to B cells and less often involves T or NK cells. The clinical course of the disease is milder and the mean age of onset is later, at 19 years. Additionally, the US cases exhibit a progressive loss of B cells, leading to hypogammaglobulinaemia, as well as reduced NK cell numbers; patients usually die of progressive B-lymphoproliferation or incidental infection [129]. However, with the advent of whole genome sequencing, some of these B cell CAEBV cases may be ascribed to one or other of the many primary immunodeficiencies that are now known to predispose to EBV pathology, and therefore may require a separate classification [130,131].

### (b) Extranodal NK/T-cell lymphoma, nasal type

Extranodal NK/T-cell lymphoma (ENKTL) is a rare but highly aggressive type of non-Hodgkin lymphoma (NHL) associated with EBV. The lymphoma is marked by extensive necrosis and angio-invasion and usually presents in extra-nodal sites, predominantly within the upper aero-digestive tract (nasal cavity, nasopharynx, paranasal sinus and palate). Patients also present in extra-nasal sites including skin, respiratory tract, gastrointestinal tract and testis. The disease mainly occurs in adults (median age 40–50 years) and predominantly in males. Interestingly, it follows a similar geographical distribution to CAEBV, with greatest frequency in East Asia, Central/South America and Mexico where the tumour accounts for 7–10% of all NHLs and 20–30% of peripheral T cell lymphomas. Comparatively, ENKTL only accounts for up to 1% of North American or European NHL cases [132]. ENKTL follows an aggressive clinical course with an extremely poor prognosis, especially for advanced disease. One of its outstanding features is an inherent resistance to conventional anthracycline-based chemotherapy, the overall 5-year survival rate of patients treated for advanced disease being merely 6–25% [133–137]. This resistance has been ascribed, at least in part, to tumour cell expression of the Multi-Drug Resistance gene (MDR1) encoding P-glycoprotein [138], and indeed the current chemotherapy regimen has been designed accordingly. However, P-glycoprotein is only expressed in a proportion of ENKTL and next generation sequencing studies have begun to identify numerous other factors that impact on apoptosis resistance and disease progression.

Chromosomal abnormalities are commonly observed in ENKTL and deletion of 6q21 is the most frequently observed [139]. This 6q21 deletion leads to the loss of expression of several tumour suppressor genes including PRDM1, ATG5, AIM1, FOXO3 and HACE1 [140,141]. Interestingly, in malignancies without the 6q21 deletion, these same tumour suppressor genes are often subject to silencing by promoter hypermethylation, highlighting a significant role for loss/silencing of these genes in EBV+ T/NK cell malignancies. Of particular note, loss of proteins such as FOXO3 and HACE1 may contribute to the apoptosis-resistance phenotype of EBV+ T/NK LPD by, respectively, preventing the induction of the pro-apoptotic BIM and PUMA, and by impairing TNF-driven NF-κB activation. Several recent studies have also identified activating mutations in STAT3 and STAT5B in up to 15% of ENKT lymphomas [142,143] and activating mutations in JAK have been variously observed [144]. Notably, all the STAT3 and STAT5B mutations were located in the SH2 domain, which is critical for STAT activation, and resulted in increased STAT phosphorylation, robust promotion of cell growth and cell survival under IL-2 limiting concentrations.

The ENKTLs exhibit a Latency II gene expression profile (table 1) with LMP1 expression being a defining feature of these malignancies. Gene expression profiling of the tumour has highlighted the activation of several oncogenic pathways including NF-κB, MAPK and JAK-STAT [140]. Mirroring the situation in several EBV-associated B lymphomas, constitutive activation of NF-κB and its downstream pathways has been proposed to be pivotal to the pathogenesis and progression of T/NK cell disease. In that context, hypo-cytokinemia is a hallmark of EBV-infected T cells, and this has been associated with NF-κB activation driven by the viral LMP1. In both *in vitro* and ex-vivo assays, when ectopically expressed in T cells, LMP1 recruits TRAF2/5 to its cytoplasmic tail and thereby activates downstream NF-κB signalling, resulting in suppression of the SAP/SH2D1A gene expression at the transcriptional level and subsequent upregulation of the Th1 cytokines TNF-α and IFN-γ [145]. At the same time, LMP1 protects those T cells from TNF-α-induced apoptosis by suppressing TNF-receptor expression and by recruiting TRADD to the LMP1 cytoplasmic tail, resulting in inhibition of the downstream caspase cascade.

One of the most intriguing and long-standing questions regarding EBV-associated T/NK cell diseases concerns EBV's means of access into the T and NK cell lineages. Mature T and NK cells are totally resistant to *in vitro* infection and indeed lack the major receptor (CD21) through which the virus infects B cells. Various lines of evidence are now beginning to emerge which allude to the answer. We previously demonstrated the presence of EBV in both the T cell and NK cell populations in individuals with EBV-driven haemophagocytic lymphohistiocytosis (HLH) [146] and, more recently, CAEBV (unpublished), raising the possibility of EBV infection of a common T/NK cell precursor. Subsequent sequence analysis of T cell and NK cell CAEBV by the Kimura group also identified individuals with EBV infection of both T cells and NK cells. More importantly, they found that both infected cell populations carried the same somatic mutation marking their common origin, thereby providing the strongest evidence yet for EBV infecting a common haematopoietic T/NK cell precursor (17th International Symposium on EBV and its associated diseases, Zurich 2016). Finally, studying the differentiation of human haematopoietic stem cells into T cells and NK cells *in vitro*, we have demonstrated the expression of the CD21 receptor before the point of T and NK lineage commitment and have successfully infected these cells with EBV (C Shannon-Lowe, unpublished). With this system, we can now begin to examine how EBV drives the transformation of T cells and NK cells into LPD lesions and ultimately into the malignancies described above.

## 7. Summary

A brief glimpse back at table 1 is sufficient to appreciate the unusually wide range of EBV-associated lymphomas, each with its own distinct pathogenetic pathway. While the ‘virus as innocent passenger’ argument cannot be completely refuted, for each of these lymphomas the presence of an active virus genome in every tumour cell constitutes strong circumstantial evidence of an aetiological role. Furthermore, where the same tumour type can occur in EBV-negative form (as it can in BL, HL and DLBCL), cytogenetic and increasingly whole genome analysis shows that where the virus is absent the spectrum of cellular genetic changes is wider and more complex than in the EBV-positive cases. This argues that, on the multi-step path to malignancy, complementation by the virus can render some genetic aberrations redundant, an idea that is being progressively reinforced as more is learnt about EBV's engagement with key cellular pathways governing cell growth and survival.

The differences in EBV gene expression between these tumour types very likely reflect the virus' different aetiologic roles. In BL, where EBV latent protein expression is usually restricted to the virus genome maintenance protein EBNA1, the tumour phenotype appears to be dominated by independent translocation/mutation events in the cell genome. Crucially, these lead to hyper-expression of two proteins, c-MYC and TCF3, with particular functions at key stages of germinal centroblast proliferation. Strong evidence suggests that EBV's role is complementary to these effects; that is, anti-apoptotic rather than growth-promoting. While the anti-apoptotic effects of Latency I infection are subtle, they nevertheless appear to be sufficient to give EBV-positive GC cells an advantage in the evolution to malignancy [32]. Importantly, switching to an LMP1-positive Latency II or III infection could offer greater protection; however such cells would cease to proliferate because, as many *in vitro* studies have shown [147,148], LMP1-driven signalling is incompatible with the c-MYC-driven (NF-κB-silent, non-inflammatory) gene expression programme that is crucial to BL cell growth.

The contrast between BL and the other main EBV-associated lymphomas is therefore stark, even though virus-mediated apoptosis protection remains a key feature. Thus, in EBV-positive HL, DLBCL and the T/NKL, there is constitutive expression of LMP1 leading to activation of NF-κB and JAK/STAT signalling. These pathways are thought to be influential in at least two respects: firstly, protection against apoptosis as described, and secondly the activation of autocrine/paracrine signals that appear to promote tumour growth and possibly also immune evasion. In this respect, both HL and the T/NKLs are tumours with a particularly heavy inflammatory environment, yet one that appears to favour tumour progression. Importantly, this link between inflammation and EBV-associated oncogenesis also extends to EBV-positive tumours of epithelial origin, namely nasopharyngeal and gastric carcinoma, and its significance is discussed further elsewhere [51]. Recent work on these carcinomas has also highlighted yet another activity of EBV that may have general relevance but has yet to be fully explored in the virus-associated lymphomas: that is the link between EBV and the tumour epigenotype. Thus a striking finding from the whole genomic analysis of gastric carcinomas was that the 10% of these tumours that are EBV-positive form a molecularly distinct subset with unusually heavily methylated epigenomes [149]. This chimes with studies in both B cell and epithelial cell systems showing upregulation of the cellular DNA methyltransferases DNMT1, DNMT3A and DNMT3B by LMP1 and LMP2 [150,151]; interestingly even transient EBV infection of epithelial cells reportedly leaves permanent epigenetic marks [152]. As we have seen in the genesis of EBV-associated lymphomas, there are several examples where the influence of key tumour suppressor genes may be lost either by mutation or by epigenetic silencing. If, for example, such silencing is more common in the EBV-positive versus EBV-negative forms of B cell lymphoma, this will reinforce the idea of EBV-induced epigenetic modification as contributing to virus oncogenicity.

Finally, there is the role of EBV as a growth-transforming agent. As is clear from the example of B-LPD, EBV can be directly oncogenic in the context of the T cell-compromised host. Whether these B-LPD lesions, which may be oligoclonal or monoclonal, are truly malignant seems largely a semantic argument since, if uncontrolled, they rapidly progress to kill the host. Crucially however, whether such growth-transforming events also represent necessary early stages of BL, HL or DLBCL pathogenesis remains an open question. While the connection appears distant in both BL and HL, it is closer in the context of DLBCL. Thus, some DLBCLs (particularly those arising in the elderly or in the setting of chronic inflammation) appear to have acquired additional genetic aberrations indicative of malignancy yet retain Latency III infection, as if they had evolved directly from growth-transformed foci. Ironically, the relationship between pre-neoplastic virus-driven LPD and lymphoma seems to be clearest in the atypical context of T/NK lineage infection where some CAEBV patients progress quite rapidly to EBV-positive T/NK lymphoma, although clonal evolution from T/NK-LPD lesion to lymphoma has not yet been formally documented by molecular studies. Interestingly the T/NK-LPD cells accumulating in the blood of CAEBV patients display a Latency II form of infection and readily expand *in vitro* with exogenous cytokine support. This suggests that EBV can initiate proliferation in this unnatural target cell type, albeit without using its classical B cell-growth transforming programme, and that such cells are then at high risk of further cellular genetic changes leading to malignancy. The example only serves to re-emphasize the enormous versatility of EBV as a lymphomagenic agent and the need for further research to resolve the many as-yet-unanswered questions posed by this virus and its fascinating links to human cancer.

## Data accessibility

This article has no additional data.

## References

[1] Young LS, Yap LF, Murray PG 2016 Epstein-Barr virus: more than 50 years old and still providing surprises. Nat. Rev. Cancer 16, 789–802. (10.1038/nrc.2016.92) 27687982

[2] Thorley-Lawson DA, Hawkins JB, Tracy SI, Shapiro M 2013 The pathogenesis of Epstein-Barr virus persistent infection. Curr. Opin. Virol. 3, 227–232. (10.1016/j.coviro.2013.04.005) 23683686 PMC3789532

[3] Hislop AD, Taylor GS, Sauce D, Rickinson AB 2007 Cellular responses to viral infection in humans: lessons from Epstein-Barr virus. Annu. Rev. Immunol. 25, 587–617. (10.1146/annurev.immunol.25.022106.141553) 17378764

[4] Hislop AD et al. 2005 Tonsillar homing of Epstein-Barr virus-specific CD8^+^ T cells and the virus-host balance. J. Clin. Invest. 115, 2546–2555. (10.1172/JCI24810) 16110323 PMC1187932

[5] Woon HG et al. 2016 Compartmentalization of total and virus-specific tissue-resident memory CD8^+^ T cells in human lymphoid organs. PLoS Pathog. 12, e1005799 (10.1371/journal.ppat.1005799) 27540722 PMC4991796

[6] Gottschalk S, Rooney CM, Heslop HE 2005 Post-transplant lymphoproliferative disorders. Annu. Rev. Med. 56, 29–44. (10.1146/annurev.med.56.082103.104727) 15660500

[7] Basso K, Dalla-Favera R 2015 Germinal centres and B cell lymphomagenesis. Nat. Rev. Immunol. 15, 172–184. (10.1038/nri3814) 25712152

[8] Timms JM, Bell A, Flavell JR, Murray PG, Rickinson AB, Traverse-Glehen A, Berger F, Delecluse HJ 2003 Target cells of Epstein-Barr-virus (EBV)-positive post-transplant lymphoproliferative disease: similarities to EBV-positive Hodgkin's lymphoma. Lancet 361, 217–223. (10.1016/S0140-6736(03)12271-4) 12547545

[9] Morscio J, Finalet Ferreiro J, Vander Borght S, Bittoun E, Gheysens O, Dierickx D, Verhoef G, Wlodarska I, Tousseyn T 2017 Identification of distinct subgroups of EBV-positive post-transplant diffuse large B-cell lymphoma. Mod. Pathol. 30, 370–381. (10.1038/modpathol.2016.199) 28059091

[10] Swerdlow SH et al. 2016 The 2016 revision of the World Health Organization classification of lymphoid neoplasms. Blood 127, 2375–2390. (10.1182/blood-2016-01-643569) 26980727 PMC4874220

[11] Molyneux EM, Rochford R, Griffin B, Newton R, Jackson G, Menon G, Harrison CJ, Israels T, Bailey S 2012 Burkitt's lymphoma. Lancet 379, 1234–1244. (10.1016/S0140-6736(11)61177-X) 22333947

[12] Moormann AM, Bailey JA 2016 Malaria—how this parasitic infection aids and abets EBV-associated Burkitt lymphomagenesis. Curr. Opin. Virol. 20, 78–84. (10.1016/j.coviro.2016.09.006) 27689909 PMC5102755

[13] Queiroga EM, Gualco G, Weiss LM, Dittmer DP, Araujo I, Klumb CE, Harrington WJ Jr, Bacchi CE 2008 Burkitt lymphoma in Brazil is characterized by geographically distinct clinicopathologic features. Am. J. Clin. Pathol. 130, 946–956. (10.1309/AJCP64YOHAWLUMPK) 19019773 PMC2866005

[14] Guech-Ongey M, Simard EP, Anderson WF, Engels EA, Bhatia K, Devesa SS, Mbulaiteye SM 2010 AIDS-related Burkitt lymphoma in the United States: what do age and CD4 lymphocyte patterns tell us about etiology and/or biology? Blood 116, 5600–5604. (10.1182/blood-2010-03-275917) 20813897 PMC3031406

[15] Clifford GM et al. 2005 Cancer risk in the Swiss HIV Cohort Study: associations with immunodeficiency, smoking, and highly active antiretroviral therapy. J. Natl Cancer Inst. 97, 425–432. (10.1093/jnci/dji072) 15770006

[16] Carroll V, Garzino-Demo A 2015 HIV-associated lymphoma in the era of combination antiretroviral therapy: shifting the immunological landscape. Pathog. Dis. 73, 44 (10.1093/femspd/ftv044) PMC4607737 26121984

[17] Arvey A et al. 2015 The tumor virus landscape of AIDS-related lymphomas. Blood 125, e14–e22. (10.1182/blood-2014-11-599951) 25827832 PMC4432014

[18] Hummel M et al. 2006 A biologic definition of Burkitt's lymphoma from transcriptional and genomic profiling. N. Engl. J. Med. 354, 2419–2430. (10.1056/NEJMoa055351) 16760442

[19] Dave SS et al. 2006 Molecular diagnosis of Burkitt's lymphoma. N. Engl. J. Med. 354, 2431–2442. (10.1056/NEJMoa055759) 16760443

[20] Guikema JE, Schuuring E, Kluin PM 2008 Structure and consequences of IGH switch breakpoints in Burkitt lymphoma. J. Natl Cancer Inst. Monogr. 2008, 32–36. (10.1093/jncimonographs/lgn020) 18647999

[21] Allday MJ 2009 How does Epstein-Barr virus (EBV) complement the activation of Myc in the pathogenesis of Burkitt's lymphoma? Semin. Cancer Biol. 19, 366–376. (10.1016/j.semcancer.2009.07.007) 19635566 PMC3770905

[22] Scheller H, Tobollik S, Kutzera A, Eder M, Unterlehberg J, Pfeil I, Jungnickel B 2010 c-Myc overexpression promotes a germinal center-like program in Burkitt's lymphoma. Oncogene 29, 888–897. (10.1038/onc.2009.377) 19881537

[23] Lindstrom MS, Wiman KG 2002 Role of genetic and epigenetic changes in Burkitt lymphoma. Semin. Cancer Biol. 12, 381–387. (10.1016/S1044-579X(02)00058-5) 12191637

[24] Schmitz R et al. 2012 Burkitt lymphoma pathogenesis and therapeutic targets from structural and functional genomics. Nature 490, 116–120. (10.1038/nature11378) 22885699 PMC3609867

[25] Love C et al. 2012 The genetic landscape of mutations in Burkitt lymphoma. Nat. Genet. 44, 1321–1325. (10.1038/ng.2468) 23143597 PMC3674561

[26] Richter J et al. 2012 Recurrent mutation of the ID3 gene in Burkitt lymphoma identified by integrated genome, exome and transcriptome sequencing. Nat. Genet. 44, 1316–1320. (10.1038/ng.2469) 23143595

[27] Abate F et al. 2015 Distinct viral and mutational spectrum of endemic Burkitt lymphoma. PLoS Pathog. 11, e1005158 (10.1371/journal.ppat.1005158) 26468873 PMC4607508

[28] Giulino-Roth L et al. 2012 Targeted genomic sequencing of pediatric Burkitt lymphoma identifies recurrent alterations in antiapoptotic and chromatin-remodeling genes. Blood 120, 5181–5184. (10.1182/blood-2012-06-437624) 23091298 PMC3537311

[29] Schmitz R, Ceribelli M, Pittaluga S, Wright G, Staudt LM 2014 Oncogenic mechanisms in Burkitt lymphoma. Cold Spring Harb. Perspect. Med. 4, a014282 (10.1101/cshperspect.a014282) 24492847 PMC3904095

[30] Sander S et al. 2012 Synergy between PI3 K signaling and MYC in Burkitt lymphomagenesis. Cancer Cell 22, 167–179. (10.1016/j.ccr.2012.06.012) 22897848 PMC3432451

[31] Thorley-Lawson DA, Allday MJ 2008 The curious case of the tumour virus: 50 years of Burkitt's lymphoma. Nat. Rev. Microbiol. 6, 913–924. (10.1038/nrmicro2015) 19008891

[32] Rowe M, Fitzsimmons L, Bell AI 2014 Epstein-Barr virus and Burkitt lymphoma. Chin. J. Cancer 33, 609–619. (10.5732/cjc.014.10190) 25418195 PMC4308657

[33] Tierney RJ, Shannon-Lowe CD, Fitzsimmons L, Bell AI, Rowe M 2015 Unexpected patterns of Epstein-Barr virus transcription revealed by a high throughput PCR array for absolute quantification of viral mRNA. Virology 474, 117–130. (10.1016/j.virol.2014.10.030) 25463610 PMC4266535

[34] Komano J, Sugiura M, Takada K 1998 Epstein-Barr virus contributes to the malignant phenotype and to apoptosis resistance in Burkitt's lymphoma cell line Akata. J. Virol. 72, 9150–9156. 9765461 10.1128/jvi.72.11.9150-9156.1998 PMC110333

[35] Nanbo A, Takada K 2002 The role of Epstein-Barr virus-encoded small RNAs (EBERs) in oncogenesis. Rev. Med. Virol. 12, 321–326. (10.1002/rmv.363) 12211044

[36] Tempera I et al. 2015 Identification of MEF2B, EBF1, and IL6R as direct gene targets of Epstein-Barr virus (EBV) nuclear antigen 1 critical for EBV-infected B-lymphocyte survival. J. Virol. 90, 345–355. (10.1128/JVI.02318-15) 26468528 PMC4702570

[37] Lu J, Murakami M, Verma SC, Cai Q, Haldar S, Kaul R, Wasik MA, Middeldorp J, Robertson ES 2011 Epstein-Barr virus nuclear antigen 1 (EBNA1) confers resistance to apoptosis in EBV-positive B-lymphoma cells through up-regulation of survivin. Virology 410, 64–75. (10.1016/j.virol.2010.10.029) 21093004 PMC4287362

[38] Dheekollu J, Malecka K, Wiedmer A, Delecluse HJ, Chiang AK, Altieri DC, Messick TE, Lieberman PM 2017 Carcinoma-risk variant of EBNA1 deregulates Epstein-Barr virus episomal latency. Oncotarget 8, 7248–7264. (10.18632/oncotarget.14540) 28077791 PMC5352318

[39] Frappier L 2012 Contributions of Epstein-Barr nuclear antigen 1 (EBNA1) to cell immortalization and survival. Viruses 4, 1537–1547. (10.3390/v4091537) 23170171 PMC3499818

[40] Klinke O, Feederle R, Delecluse HJ 2014 Genetics of Epstein-Barr virus microRNAs. Semin. Cancer Biol. 26, 52–59. (10.1016/j.semcancer.2014.02.002) 24602823

[41] Vereide DT, Seto E, Chiu YF, Hayes M, Tagawa T, Grundhoff A, Hammerschmidt W, Sugden B 2014 Epstein-Barr virus maintains lymphomas via its miRNAs. Oncogene 33, 1258–1264. (10.1038/onc.2013.71) 23503461 PMC3690170

[42] Piccaluga PP et al. 2016 Virus-encoded microRNA contributes to the molecular profile of EBV-positive Burkitt lymphomas. Oncotarget 7, 224–240. (10.18632/oncotarget.4399) 26325594 PMC4807994

[43] Ambrosio MR et al. 2014 The Epstein Barr-encoded BART-6-3p microRNA affects regulation of cell growth and immuno response in Burkitt lymphoma. Infect. Agent. Cancer 9, 12 (10.1186/1750-9378-9-12) 24731550 PMC4005456

[44] Harold C, Cox D, Riley KJ 2016 Epstein-Barr viral microRNAs target caspase 3. Virol. J. 13, 145 (10.1186/s12985-016-0602-7) 27565721 PMC5002152

[45] Robbiani DF et al. 2008 AID is required for the chromosomal breaks in c-myc that lead to c-myc/IgH translocations. Cell 135, 1028–1038. (10.1016/j.cell.2008.09.062) 19070574 PMC2713603

[46] Epeldegui M, Hung YP, McQuay A, Ambinder RF, Martinez-Maza O 2007 Infection of human B cells with Epstein-Barr virus results in the expression of somatic hypermutation-inducing molecules and in the accrual of oncogene mutations. Mol. Immunol. 44, 934–942. (10.1016/j.molimm.2006.03.018) 16730063

[47] Kalchschmidt JS, Bashford-Rogers R, Paschos K, Gillman AC, Styles CT, Kellam P, Allday MJ 2016 Epstein-Barr virus nuclear protein EBNA3C directly induces expression of AID and somatic mutations in B cells. J. Exp. Med. 213, 921–928. (10.1084/jem.20160120) 27217538 PMC4886369

[48] Wood CD et al. 2016 MYC activation and BCL2L11 silencing by a tumour virus through the large-scale reconfiguration of enhancer-promoter hubs. Elife 5, e18270 (10.7554/eLife.18270) 27490482 PMC5005034

[49] Torgbor C, Awuah P, Deitsch K, Kalantari P, Duca KA, Thorley-Lawson DA 2014 A multifactorial role for *P. falciparum* malaria in endemic Burkitt's lymphoma pathogenesis. PLoS Pathog. 10, e1004170 (10.1371/journal.ppat.1004170) 24874410 PMC4038605

[50] Robbiani DF et al. 2015 *Plasmodium* infection promotes genomic instability and AID-dependent B cell lymphoma. Cell 162, 727–737. (10.1016/j.cell.2015.07.019) 26276629 PMC4538708

[51] Rickinson AB 2014 Co-infections, inflammation and oncogenesis: future directions for EBV research. Semin. Cancer Biol. 26, 99–115. (10.1016/j.semcancer.2014.04.004) 24751797

[52] Magrath I 2012 Epidemiology: clues to the pathogenesis of Burkitt lymphoma. Br. J. Haematol. 156, 744–756. (10.1111/j.1365-2141.2011.09013.x) 22260300

[53] Amato T et al. 2016 Clonality analysis of immunoglobulin gene rearrangement by next-generation sequencing in endemic Burkitt lymphoma suggests antigen drive activation of BCR as opposed to sporadic Burkitt lymphoma. Am. J. Clin. Pathol. 145, 116–127. (10.1093/ajcp/aqv011) 26712879 PMC4778259

[54] Piccaluga PP et al. 2011 Gene expression analysis uncovers similarity and differences among Burkitt lymphoma subtypes. Blood 117, 3596–3608. (10.1182/blood-2010-08-301556) 21245480

[55] Kaymaz Y, Oduor CI, Yu H, Otieno JA, Ong'echa JM, Moormann AM, Bailey JA 2017 Comprehensive transcriptome and mutational profiling of endemic Burkitt lymphoma reveals EBV type-specific differences. Mol. Cancer Res. 15, 499 (10.1158/1541-7786.MCR-16-0305) PMC5471630 28465297

[56] Pileri SA et al. 2002 Hodgkin's lymphoma: the pathologist's viewpoint. J. Clin. Pathol. 55, 162–176. (10.1136/jcp.55.3.162) 11896065 PMC1769601

[57] Mathas S, Hartmann S, Kuppers R 2016 Hodgkin lymphoma: pathology and biology. Semin. Hematol. 53, 139–147. (10.1053/j.seminhematol.2016.05.007) 27496304

[58] Aldinucci D, Celegato M, Casagrande N 2016 Microenvironmental interactions in classical Hodgkin lymphoma and their role in promoting tumor growth, immune escape and drug resistance. Cancer Lett. 380, 243–252. (10.1016/j.canlet.2015.10.007) 26474544

[59] Glaser SL et al. 1997 Epstein-Barr virus-associated Hodgkin's disease: epidemiologic characteristics in international data. Int. J. Cancer 70, 375–382. (10.1002/(SICI)1097-0215(19970207)70:4%3C375::AID-IJC1%3E3.0.CO;2-T) 9033642

[60] Cesarman E 2013 Pathology of lymphoma in HIV. Curr. Opin. Oncol. 25, 487–494. (10.1097/01.cco.0000432525.70099.a4) 23942293 PMC4126602

[61] Kuppers R, Rajewsky K, Zhao M, Simons G, Laumann R, Fischer R, Hansmann ML 1994 Hodgkin disease: Hodgkin and Reed-Sternberg cells picked from histological sections show clonal immunoglobulin gene rearrangements and appear to be derived from B cells at various stages of development. Proc. Natl Acad. Sci. USA 91, 10 962–10 966. (10.1073/pnas.91.23.10962) PMC45146 7971992

[62] Schwering I et al. 2003 Loss of the B-lineage-specific gene expression program in Hodgkin and Reed-Sternberg cells of Hodgkin lymphoma. Blood 101, 1505–1512. (10.1182/blood-2002-03-0839) 12393731

[63] Hertel CB, Zhou XG, Hamilton-Dutoit SJ, Junker S 2002 Loss of B cell identity correlates with loss of B cell-specific transcription factors in Hodgkin/Reed-Sternberg cells of classical Hodgkin lymphoma. Oncogene 21, 4908–4920. (10.1038/sj.onc.1205629) 12118370

[64] Guan H, Xie L, Wirth T, Ushmorov A 2016 Repression of TCF3/E2A contributes to Hodgkin lymphomagenesis. Oncotarget 7, 36 854–36 864. (10.18632/oncotarget.9210) PMC5095044 27166193

[65] Cozen W et al. 2014 A meta-analysis of Hodgkin lymphoma reveals 19p13.3 TCF3 as a novel susceptibility locus. Nat. Commun. 5, 3856 (10.1038/ncomms4856) 24920014 PMC4055950

[66] Farrell K, Jarrett RF 2011 The molecular pathogenesis of Hodgkin lymphoma. Histopathology 58, 15–25. (10.1111/j.1365-2559.2010.03705.x) 21261680

[67] Kanzler H, Kuppers R, Hansmann ML, Rajewsky K 1996 Hodgkin and Reed-Sternberg cells in Hodgkin's disease represent the outgrowth of a dominant tumor clone derived from (crippled) germinal center B cells. J. Exp. Med. 184, 1495–1505. (10.1084/jem.184.4.1495) 8879220 PMC2192840

[68] Weniger MA, Kuppers R 2016 NF-κB deregulation in Hodgkin lymphoma. Semin. Cancer Biol. 39, 32–39. (10.1016/j.semcancer.2016.05.001) 27221964

[69] Joos S et al. 2000 Genomic imbalances including amplification of the tyrosine kinase gene JAK2 in CD30^+^ Hodgkin cells. Cancer Res. 60, 549–552. 10676635

[70] Reichel J et al. 2015 Flow sorting and exome sequencing reveal the oncogenome of primary Hodgkin and Reed-Sternberg cells. Blood 125, 1061–1072. (10.1182/blood-2014-11-610436) 25488972

[71] Liu Y et al. 2014 The mutational landscape of Hodgkin lymphoma cell lines determined by whole-exome sequencing. Leukemia 28, 2248–2251. (10.1038/leu.2014.201) 24947018

[72] Roemer MG et al. 2016 Classical Hodgkin lymphoma with reduced beta2M/MHC class I expression is associated with inferior outcome independent of 9p24.1 status. Cancer Immunol. Res. 4, 910–916. (10.1158/2326-6066.CIR-16-0201) 27737878 PMC5210180

[73] Vockerodt M et al. 2008 The Epstein-Barr virus oncoprotein, latent membrane protein-1, reprograms germinal centre B cells towards a Hodgkin's Reed-Sternberg-like phenotype. J. Pathol. 216, 83–92. (10.1002/path.2384) 18566961

[74] Vrzalikova K et al. 2011 Down-regulation of BLIMP1alpha by the EBV oncogene, LMP-1, disrupts the plasma cell differentiation program and prevents viral replication in B cells: implications for the pathogenesis of EBV-associated B-cell lymphomas. Blood 117, 5907–5917. (10.1182/blood-2010-09-307710) 21411757 PMC3293751

[75] Vockerodt M, Wei W, Nagy E, Prouzova Z, Schrader A, Kube D, Rowe M, Woodman CB, Murray PG 2013 Suppression of the LMP2A target gene, EGR-1, protects Hodgkin's lymphoma cells from entry to the EBV lytic cycle. J. Pathol. 230, 399–409. (10.1002/path.4198) 23592216

[76] Brauninger A, Schmitz R, Bechtel D, Renne C, Hansmann ML, Kuppers R 2006 Molecular biology of Hodgkin's and Reed/Sternberg cells in Hodgkin's lymphoma. Int. J. Cancer 118, 1853–1861. (10.1002/ijc.21716) 16385563

[77] Chaganti S, Bell AI, Pastor NB, Milner AE, Drayson M, Gordon J, Rickinson AB 2005 Epstein-Barr virus infection in vitro can rescue germinal center B cells with inactivated immunoglobulin genes. Blood 106, 4249–4252. (10.1182/blood-2005-06-2327) 16123211

[78] Schmitz R et al. 2009 TNFAIP3 (A20) is a tumor suppressor gene in Hodgkin lymphoma and primary mediastinal B cell lymphoma. J. Exp. Med. 206, 981–989. (10.1084/jem.20090528) 19380639 PMC2715030

[79] Renne C et al. 2007 The aberrant coexpression of several receptor tyrosine kinases is largely restricted to EBV-negative cases of classical Hodgkin's lymphoma. Int. J. Cancer 120, 2504–2509. (10.1002/ijc.22511) 17330841

[80] Leonard S, Wei W, Anderton J, Vockerodt M, Rowe M, Murray PG, Woodman CB 2011 Epigenetic and transcriptional changes which follow Epstein-Barr virus infection of germinal center B cells and their relevance to the pathogenesis of Hodgkin's lymphoma. J. Virol. 85, 9568–9577. (10.1128/JVI.00468-11) 21752916 PMC3165764

[81] Alizadeh AA et al. 2000 Distinct types of diffuse large B-cell lymphoma identified by gene expression profiling. Nature 403, 503–511. (10.1038/35000501) 10676951

[82] Oyama T et al. 2003 Senile EBV+ B-cell lymphoproliferative disorders: a clinicopathologic study of 22 patients. Am. J. Surg. Pathol. 27, 16–26. (10.1097/00000478-200301000-00003) 12502924

[83] Shimoyama Y, Yamamoto K, Asano N, Oyama T, Kinoshita T, Nakamura S 2008 Age-related Epstein-Barr virus-associated B-cell lymphoproliferative disorders: special references to lymphomas surrounding this newly recognized clinicopathologic disease. Cancer Sci. 99, 1085–1091. (10.1111/j.1349-7006.2008.00813.x) 18429953 PMC11159301

[84] Cohen M, De Matteo E, Narbaitz M, Carreno FA, Preciado MV, Chabay PA 2013 Epstein-Barr virus presence in pediatric diffuse large B-cell lymphoma reveals a particular association and latency patterns: analysis of viral role in tumor microenvironment. Int. J. Cancer 132, 1572–1580. (10.1002/ijc.27845) 22987474

[85] Cohen M, Narbaitz M, Metrebian F, De Matteo E, Preciado MV, Chabay PA 2014 Epstein-Barr virus-positive diffuse large B-cell lymphoma association is not only restricted to elderly patients. Int. J. Cancer 135, 2816–2824. (10.1002/ijc.28942) 24789501

[86] Cardenas D et al. 2015 Epstein-Barr virus-specific CD8^+^ T lymphocytes from diffuse large B cell lymphoma patients are functionally impaired. Clin. Exp. Immunol. 182, 173–183. (10.1111/cei.12682) 26174440 PMC4608507

[87] Ok CY, Papathomas TG, Medeiros LJ, Young KH 2013 EBV-positive diffuse large B-cell lymphoma of the elderly. Blood 122, 328–340. (10.1182/blood-2013-03-489708) 23649469 PMC3779382

[88] White RE et al. 2012 EBNA3B-deficient EBV promotes B cell lymphomagenesis in humanized mice and is found in human tumors. J. Clin. Invest. 122, 1487–1502. (10.1172/JCI58092) 22406538 PMC3314448

[89] Sebastian E et al. 2016 High-resolution copy number analysis of paired normal-tumor samples from diffuse large B cell lymphoma. Ann. Hematol. 95, 253–262. (10.1007/s00277-015-2552-3) 26573278

[90] Shaffer AL 3rd, Young RM, Staudt LM 2012 Pathogenesis of human B cell lymphomas. Annu. Rev. Immunol. 30, 565–610. (10.1146/annurev-immunol-020711-075027) 22224767 PMC7478144

[91] Mandelbaum J et al. 2010 BLIMP1 is a tumor suppressor gene frequently disrupted in activated B cell-like diffuse large B cell lymphoma. Cancer Cell 18, 568–579. (10.1016/j.ccr.2010.10.030) 21156281 PMC3030476

[92] Pasqualucci L et al. 2006 Inactivation of the PRDM1/BLIMP1 gene in diffuse large B cell lymphoma. J. Exp. Med. 203, 311–317. (10.1084/jem.20052204) 16492805 PMC2118216

[93] Tam W, Gomez M, Chadburn A, Lee JW, Chan WC, Knowles DM 2006 Mutational analysis of PRDM1 indicates a tumor-suppressor role in diffuse large B-cell lymphomas. Blood 107, 4090–4100. (10.1182/blood-2005-09-3778) 16424392

[94] Lam LT et al. 2005 Small molecule inhibitors of IκB kinase are selectively toxic for subgroups of diffuse large B-cell lymphoma defined by gene expression profiling. Clin. Cancer Res. 11, 28–40. 15671525

[95] Kato H et al. 2014 Gene expression profiling of Epstein-Barr virus-positive diffuse large B-cell lymphoma of the elderly reveals alterations of characteristic oncogenetic pathways. Cancer Sci. 105, 537–544. (10.1111/cas.12389) 24581222 PMC4317839

[96] Loong F et al. 2010 Diffuse large B-cell lymphoma associated with chronic inflammation as an incidental finding and new clinical scenarios. Mod. Pathol. 23, 493–501. (10.1038/modpathol.2009.168) 20062008

[97] Sanchez-Gonzalez B, Garcia M, Montserrat F, Sanchez M, Angona A, Solano A, Salar A 2013 Diffuse large B-cell lymphoma associated with chronic inflammation in metallic implant. J. Clin. Oncol. 31, e148–e151. (10.1200/JCO.2012.42.8250) 23401446

[98] Cheuk W, Chan AC, Chan JK, Lau GT, Chan VN, Yiu HH 2005 Metallic implant-associated lymphoma: a distinct subgroup of large B-cell lymphoma related to pyothorax-associated lymphoma? Am. J. Surg. Pathol. 29, 832–836. (10.1097/01.pas.0000157747.10967.f4) 15897752

[99] Copie-Bergman C, Niedobitek G, Mangham DC, Selves J, Baloch K, Diss TC, Knowles DN, Delsol G, Isaacson PG 1997 Epstein-Barr virus in B-cell lymphomas associated with chronic suppurative inflammation. J. Pathol. 183, 287–292. (10.1002/(SICI)1096-9896(199711)183:3%3C287::AID-PATH932%3E3.0.CO;2-Q) 9422983

[100] Kanno H, Naka N, Yasunaga Y, Iuchi K, Yamauchi S, Hashimoto M, Aozasa K 1997 Production of the immunosuppressive cytokine interleukin-10 by Epstein-Barr-virus-expressing pyothorax-associated lymphoma: possible role in the development of overt lymphoma in immunocompetent hosts. Am. J. Pathol. 150, 349–357. 9006350 PMC1858513

[101] Kanno H, Naka N, Yasunaga Y, Aozasa K 1997 Role of an immunosuppressive cytokine, interleukin-10, in the development of pyothorax-associated lymphoma. Leukemia 11(Suppl. 3), 525–526. 9209445

[102] Kanno H, Yasunaga Y, Iuchi K, Yamauchi S, Tatekawa T, Sugiyama H, Aozasa K 1996 Interleukin-6-mediated growth enhancement of cell lines derived from pyothorax-associated lymphoma. Lab. Invest. 75, 167–173. 8765317

[103] Kanno H, Ohsawa M, Hashimoto M, Iuchi K, Nakajima Y, Aozasa K 1999 HLA-A alleles of patients with pyothorax-associated lymphoma: anti-Epstein-Barr virus (EBV) host immune responses during the development of EBV latent antigen-positive lymphomas. Int. J. Cancer 82, 630–634. (10.1002/(SICI)1097-0215(19990827)82:5%3C630::AID-IJC2%3E3.0.CO;2-D) 10417757

[104] Kanno H, Nakatsuka S, Iuchi K, Aozasa K 2000 Sequences of cytotoxic T-lymphocyte epitopes in the Epstein-Barr virus (EBV) nuclear antigen-3B gene in a Japanese population with or without EBV-positive lymphoid malignancies. Int. J. Cancer 88, 626–632. (10.1002/1097-0215(20001115)88:4%3C626::AID-IJC17%3E3.0.CO;2-Q) 11058881

[105] Nishiu M et al. 2004 Distinct pattern of gene expression in pyothorax-associated lymphoma (PAL), a lymphoma developing in long-standing inflammation. Cancer Sci. 95, 828–834. (10.1111/j.1349-7006.2004.tb02189.x) 15504251 PMC11159202

[106] Gibson TM, Morton LM, Shiels MS, Clarke CA, Engels EA 2014 Risk of non-Hodgkin lymphoma subtypes in HIV-infected people during the HAART era: a population-based study. AIDS 28, 2313–2318. (10.1097/QAD.0000000000000428) 25111081 PMC4260326

[107] Glotz D et al. 2012 The Seville expert workshop for progress in posttransplant lymphoproliferative disorders. Transplantation 94, 784–793. (10.1097/TP.0b013e318269e64f) 22992767

[108] Dharnidharka VR, Webster AC, Martinez OM, Preiksaitis JK, Leblond V, Choquet S 2016 Post-transplant lymphoproliferative disorders. Nat. Rev. Dis. Primers 2, 15088 (10.1038/nrdp.2015.88) 27189056

[109] Ferreiro JF, Morscio J, Dierickx D, Vandenberghe P, Gheysens O, Verhoef G, Zamani M, Tousseyn T, Wlodarska I 2016 EBV-positive and EBV-negative posttransplant diffuse large B cell lymphomas have distinct genomic and transcriptomic features. Am. J. Transplant. 16, 414–425. (10.1111/ajt.13558) 26780579

[110] Morscio J et al. 2013 Gene expression profiling reveals clear differences between EBV-positive and EBV-negative posttransplant lymphoproliferative disorders. Am. J. Transplant. 13, 1305–1316. (10.1111/ajt.12196) 23489474

[111] Menter T, Juskevicius D, Alikian M, Steiger J, Dirnhofer S, Tzankov A, Naresh KN 2017 Mutational landscape of B-cell post-transplant lymphoproliferative disorders. Br. J. Haematol. 178, 341–491. (10.1111/bjh.14633) 28419429

[112] Craig FE, Johnson LR, Harvey SA, Nalesnik MA, Luo JH, Bhattacharya SD, Swerdlow SH 2007 Gene expression profiling of Epstein-Barr virus-positive and -negative monomorphic B-cell posttransplant lymphoproliferative disorders. Diagn. Mol. Pathol. 16, 158–168. (10.1097/PDM.0b013e31804f54a9) 17721324

[113] Delecluse HJ et al. 1997 Plasmablastic lymphomas of the oral cavity: a new entity associated with the human immunodeficiency virus infection. Blood 89, 1413–1420. 9028965

[114] Nador RG, Cesarman E, Knowles DM, Said JW 1995 Herpes-like DNA sequences in a body-cavity-based lymphoma in an HIV-negative patient. N. Engl. J. Med. 333, 943 (10.1056/NEJM199510053331417) 7666892

[115] Dolcetti R, Gloghini A, Caruso A, Carbone A 2016 A lymphomagenic role for HIV beyond immune suppression? Blood 127, 1403–1409. (10.1182/blood-2015-11-681411) 26773045 PMC4826146

[116] Schulz TF, Cesarman E 2015 Kaposi sarcoma-associated herpesvirus: mechanisms of oncogenesis. Curr. Opin. Virol. 14, 116–128. (10.1016/j.coviro.2015.08.016) 26431609

[117] Fan W, Bubman D, Chadburn A, Harrington WJ Jr, Cesarman E, Knowles DM 2005 Distinct subsets of primary effusion lymphoma can be identified based on their cellular gene expression profile and viral association. J. Virol. 79, 1244–1251. (10.1128/JVI.79.2.1244-1251.2005) 15613351 PMC538532

[118] Kimura H et al. 2003 Prognostic factors for chronic active Epstein-Barr virus infection. J. Infect. Dis. 187, 527–533. (10.1086/367988) 12599068

[119] Kimura H, Hoshino Y, Kanegane H, Tsuge I, Okamura T, Kawa K, Morishima T 2001 Clinical and virologic characteristics of chronic active Epstein-Barr virus infection. Blood 98, 280–286. (10.1182/blood.V98.2.280) 11435294

[120] Kimura H et al. 2005 Differences between T cell-type and natural killer cell-type chronic active Epstein-Barr virus infection. J. Infect. Dis. 191, 531–539. (10.1086/427239) 15655776

[121] Ohga S et al. 1999 Restricted diversification of T-cells in chronic active Epstein-Barr virus infection: potential inclination to T-lymphoproliferative disease. Am. J. Hematol. 61, 26–33. (10.1002/(SICI)1096-8652(199905)61:1%3C26::AID-AJH6%3E3.0.CO;2-R) 10331508

[122] Oyoshi MK et al. 2003 Preferential expansion of Vgamma9-JgammaP/Vdelta2-Jdelta3 gammadelta T cells in nasal T-cell lymphoma and chronic active Epstein-Barr virus infection. Am. J. Pathol. 162, 1629–1638. (10.1016/S0002-9440(10)64297-6) 12707047 PMC1851204

[123] Fujieda M, Wakiguchi H, Hisakawa H, Kubota H, Kurashige T 1993 Defective activity of Epstein-Barr virus (EBV) specific cytotoxic T lymphocytes in children with chronic active EBV infection and in their parents. Acta Paediatr. Jpn 35, 394–399. (10.1111/j.1442-200X.1993.tb03079.x) 8256622

[124] Sugaya N, Kimura H, Hara S, Hoshino Y, Kojima S, Morishima T, Tsurumi T, Kuzushima K 2004 Quantitative analysis of Epstein-Barr virus (EBV)-specific CD8^+^ T cells in patients with chronic active EBV infection. J. Infect. Dis. 190, 985–988. (10.1086/423285) 15295706

[125] Joncas J, Monczak Y, Ghibu F, Alfieri C, Bonin A, Ahronheim G, Rivard G 1989 Brief report: killer cell defect and persistent immunological abnormalities in two patients with chronic active Epstein-Barr virus infection. J. Med. Virol. 28, 110–117. (10.1002/jmv.1890280211) 2544675

[126] Fox CP et al. 2010 A novel latent membrane 2 transcript expressed in Epstein-Barr virus-positive NK- and T-cell lymphoproliferative disease encodes a target for cellular immunotherapy. Blood 116, 3695–3704. (10.1182/blood-2010-06-292268) 20671118 PMC2981530

[127] Wakiguchi H, Fujieda M, Matsumoto K, Ohara Y, Wakiguchi A, Kurashige T 1988 Defective killer cell activity in patients with chronic active Epstein-Barr virus infection. Acta Med. Okayama 42, 137–142. 2840801 10.18926/AMO/31028

[128] Ohga S et al. 2004 Dominant expression of interleukin-10 and transforming growth factor-beta genes in activated T-cells of chronic active Epstein-Barr virus infection. J. Med. Virol. 74, 449–458. (10.1002/jmv.20197) 15368517

[129] Cohen JI et al. 2011 Characterization and treatment of chronic active Epstein-Barr virus disease: a 28-year experience in the United States. Blood 117, 5835–5849. (10.1182/blood-2010-11-316745) 21454450 PMC3112034

[130] Cohen JI 2015 Primary immunodeficiencies associated with EBV disease. Curr. Top. Microbiol. Immunol. 390, 241–265. (10.1007/978-3-319-22822-8_10) 26424649 PMC6349415

[131] Tangye SG, Palendira U, Edwards ES 2017 Human immunity against EBV-lessons from the clinic. J. Exp. Med. 214, 269–283. (10.1084/jem.20161846) 28108590 PMC5294862

[132] Au WY 2010 Current management of nasal NK/T-cell lymphoma. Oncology 24, 352–358. 20464847

[133] Pagano L et al. 2006 NK/T-cell lymphomas ‘nasal type’: an Italian multicentric retrospective survey. Ann. Oncol. 17, 794–800. (10.1093/annonc/mdl015) 16497823

[134] Au WY et al. 2005 Clinicopathologic features and treatment outcome of mature T-cell and natural killer-cell lymphomas diagnosed according to the World Health Organization classification scheme: a single center experience of 10 years. Ann. Oncol. 16, 206–214. (10.1093/annonc/mdi037) 15668271

[135] Chauchet A et al. 2012 Complete remission after first-line radio-chemotherapy as predictor of survival in extranodal NK/T cell lymphoma. J. Hematol. Oncol. 5, 27 (10.1186/1756-8722-5-27) 22682004 PMC3416641

[136] Suzuki R et al. 2010 Prognostic factors for mature natural killer (NK) cell neoplasms: aggressive NK cell leukemia and extranodal NK cell lymphoma, nasal type. Ann. Oncol. 21, 1032–1040. (10.1093/annonc/mdp418) 19850638

[137] Lee J et al. 2006 Extranodal natural killer T-cell lymphoma, nasal-type: a prognostic model from a retrospective multicenter study. J. Clin. Oncol. 24, 612–618. (10.1200/JCO.2005.04.1384) 16380410

[138] Drenou B et al. 1997 CD3^−^ CD56^+^ non-Hodgkin's lymphomas with an aggressive behavior related to multidrug resistance. Blood 89, 2966–2974. 9108417

[139] Nakashima Y et al. 2005 Genome-wide array-based comparative genomic hybridization of natural killer cell lymphoma/leukemia: different genomic alteration patterns of aggressive NK-cell leukemia and extranodal Nk/T-cell lymphoma, nasal type. Genes Chromosomes Cancer 44, 247–255. (10.1002/gcc.20245) 16049916

[140] Jiang L et al. 2015 Exome sequencing identifies somatic mutations of DDX3X in natural killer/T-cell lymphoma. Nat. Genet. 47, 1061–1066. (10.1038/ng.3358) 26192917

[141] Huang Y, de Leval L, Gaulard P 2013 Molecular underpinning of extranodal NK/T-cell lymphoma. Best Pract. Res. Clin. Haematol. 26, 57–74. (10.1016/j.beha.2013.04.006) 23768641

[142] Quintanilla-Martinez L et al. 2001 p53 Mutations in nasal natural killer/T-cell lymphoma from Mexico: association with large cell morphology and advanced disease. Am. J. Pathol. 159, 2095–2105. (10.1016/S0002-9440(10)63061-1) 11733360 PMC1850589

[143] Kucuk C et al. 2015 Activating mutations of STAT5B and STAT3 in lymphomas derived from gammadelta-T or NK cells. Nat. Commun. 6, 6025 (10.1038/ncomms7025) 25586472 PMC7743911

[144] Koo GC et al. 2012 Janus kinase 3-activating mutations identified in natural killer/T-cell lymphoma. Cancer Discov. 2, 591–597. (10.1158/2159-8290.CD-12-0028) 22705984

[145] Chuang HC, Lay JD, Hsieh WC, Wang HC, Chang Y, Chuang SE, Su IJ 2005 Epstein-Barr virus LMP1 inhibits the expression of SAP gene and upregulates Th1 cytokines in the pathogenesis of hemophagocytic syndrome. Blood 106, 3090–3096. (10.1182/blood-2005-04-1406) 16002423

[146] Fox CP, Shannon-Lowe C, Gothard P, Kishore B, Neilson J, O'Connor N, Rowe M 2010 Epstein-Barr virus-associated hemophagocytic lymphohistiocytosis in adults characterized by high viral genome load within circulating natural killer cells. Clin. Infect. Dis. 51, 66–69. (10.1086/653424) 20504238

[147] Pajic A et al. 2001 Antagonistic effects of c-myc and Epstein-Barr virus latent genes on the phenotype of human B cells. Int. J. Cancer 93, 810–816. (10.1002/ijc.1404) 11519042

[148] Polack A et al. 1996 c-myc activation renders proliferation of Epstein-Barr virus (EBV)-transformed cells independent of EBV nuclear antigen 2 and latent membrane protein 1. Proc. Natl Acad. Sci. USA 93, 10 411–10 416. (10.1073/pnas.93.19.10411) PMC38398 8816814

[149] Cancer Genome Atlas Research Network. 2014 Comprehensive molecular characterization of gastric adenocarcinoma. Nature 513, 202–209. (10.1038/nature13480) 25079317 PMC4170219

[150] Tsai CL et al. 2006 Activation of DNA methyltransferase 1 by EBV LMP1 involves c-Jun NH(2)-terminal kinase signaling. Cancer Res. 66, 11 668–11 676. (10.1158/0008-5472.CAN-06-2194) 17178861

[151] Hino R et al. 2009 Activation of DNA methyltransferase 1 by EBV latent membrane protein 2A leads to promoter hypermethylation of PTEN gene in gastric carcinoma. Cancer Res. 69, 2766–2774. (10.1158/0008-5472.CAN-08-3070) 19339266

[152] Queen KJ, Shi M, Zhang F, Cvek U, Scott RS 2013 Epstein-Barr virus-induced epigenetic alterations following transient infection. Int. J. Cancer 132, 2076–2086. (10.1002/ijc.27893) 23047626 PMC3578144

